# Fluorescently Labeled DNA Interacts with Competence and Recombination Proteins and Is Integrated and Expressed Following Natural Transformation of Bacillus subtilis

**DOI:** 10.1128/mBio.01161-18

**Published:** 2018-09-25

**Authors:** Mirjam Boonstra, Nina Vesel, Oscar P. Kuipers

**Affiliations:** a Department of Molecular Genetics, Faculty of Science and Engineering, Groningen Biomolecular Sciences and Biotechnology Institute (GBB), University of Groningen, Groningen, the Netherlands; Max Planck Institute for Terrestrial Microbiology

**Keywords:** *Bacillus subtilis*, genetic competence, labeled DNA, microfluidics, transformation

## Abstract

During competence, Bacillus subtilis is able to take up DNA from its environment through the process of transformation. We investigated the ability of B. subtilis to take up fluorescently labeled DNA and found that it is able to take up fluorescein-dUTP-, DyLight 550-dUTP-, and DyLight 650-dUTP-labeled DNA. Transformation with labeled DNA containing an antibiotic cassette resulted in uptake of the labeled DNA and also generated antibiotic-resistant colonies. DNA is primarily taken up at the pole, as it can be seen to colocalize with ComFC, which is a component of the competence machinery. The DNA is taken up rapidly and can be seen to localize with (the actively searching form of) RecA. Colocalization with a homologous locus on the chromosome increases over time. Using microfluidics, we observed replacement of the homologous locus and subsequent expression of the integrated labeled and unlabeled DNA, although whether the integrated DNA contains labeled nucleotides needs to be determined conclusively. Integrated DNA in cells with a doubling time of 60 min is expressed on average 6 h 45 min after the addition of DNA and 4 h 45 min after the addition of fresh medium. We also found that the expression of the incoming DNA under these conditions can occur before cell division and, thus, before complete exit from the competence state. Because the competence machinery is conserved among naturally competent bacteria, this method of labeling is also suitable for studying transformation of other naturally competent bacteria.

## Abstract (precis)

We used DNA that was covalently labeled with fluorescent nucleotides to investigate the transformation process of Bacillus subtilis at the molecular level. We show that the labeled DNA colocalizes with components of the competence machinery, the chromosome, and the recombination protein RecA. Using time-lapse microscopy and microfluidics, we visualized, in real-time, the uptake of fluorescently labeled DNA. We found that under these conditions, cell division is not required for the expression of integrated DNA. Because the competence machinery is conserved in naturally competent bacteria, this method can also be used to investigate the transformation process in many other bacterial species.

## INTRODUCTION

A fascinating adaptive strategy of Bacillus subtilis is its ability to take up exogenous DNA from the environment during natural competence. Naturally competent B. subtilis contains a large multiprotein competence machinery, which is primarily located at the pole (1, 2). The process of transformation occurs through the initial binding of double-stranded DNA to ComEA and ComEC (3–6). After binding, one of the DNA strands is degraded, likely by a nuclease domain of ComEC, while the other strand is transported as single-stranded DNA (ssDNA) through the ComEC channel (5–8). B. subtilis does not have a preference for the 3′-to-5′ or the 5′-to-3′ strand, nor does it require a specific sequence to be present in order to take up exogenous DNA (9, 10). While the exogenous DNA is present at the surface of the cell, it is still accessible to DNase I, but approximately 1 to 1.5 min after the addition of DNA at 37°C, the exogenous DNA becomes resistant to DNase I and single-strand donor DNA can be retrieved from lysed cells, confirming uptake of DNA (11–13). If the foreign DNA has homology to the genomic DNA of the recipient, it can be integrated into the chromosome by homologous recombination (14). One of the main components responsible for the integration of homologous DNA into the host chromosome is RecA (15–17). During competence, RecA localizes near the competence machinery (18). Upon the addition of exogenous DNA, it forms a filamentous structure, which has been proposed to be the form it takes when actively searching for homology (18). Several studies, using fluorescent-protein fusions to Com proteins, have shown the colocalization of components of the competence machinery (1, 2, 19). The localization and interaction of competence and replication proteins have thus been relatively well characterized. Visualization of interactions of fluorescent DNA with the competence machinery has proven to be more challenging. The excellent research done on transformation of Helicobacter pylori and B. subtilis by Stingl et al. showed uptake of fluorescently labeled DNA into the periplasm of the Gram-negative H. pylori. They found that labeled DNA passes through the type IV secretion system ComB in the outer membrane. However, no transfer of labeled DNA into the cytoplasm of H. pylori was detected (20). Fluorescently labeled DNA given to B. subtilis remained sensitive to DNase I, which means that the labeled DNA was not internalized (20). Although transport of fluorescently labeled DNA into the cytoplasm of B. subtilis was not successful previously (20), we were curious as to whether different labeling methods and dyes could result in cases of uptake of fluorescent DNA by B. subtilis. We therefore determined which types of dyes are suitable for labeling and if there are any requirements with regard to the size, charge, and hydrophobicity of the dyes. First, these dyes were used to answer the questions of whether B. subtilis can take up fluorescently labeled DNA via transformation and whether cells transformed with labeled DNA containing an antibiotic resistance gene form resistant colonies. After the selection of appropriate dyes, we sought to use them to gain further biological insight. Colocalization of fluorescently labeled DNA with a component of the competence machinery, with RecA, and with the chromosome were investigated. We also wanted to know if we could follow the entire transformation process, from DNA-binding to uptake, integration, and expression. To determine the timing of the transformation process, time-lapse microscopy in combination with microfluidics was used. Specifically, we sought to answer the questions of how long it takes before DNA is integrated into the chromosome and expressed, whether cell division occurs before the expression of the integrated DNA, and whether the replacement of a specific locus on the chromosome can be visualized.

## RESULTS

### Labeled DNA was internalized and DNase I resistant.

**FIG 1:**
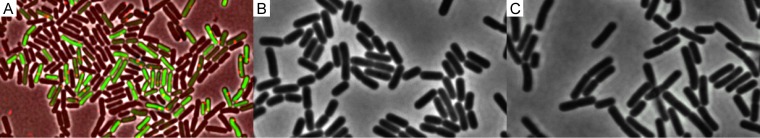
Comparison of competent and noncompetent B. subtiltis strains incubated with labeled DNA for 1 h and with DNase I for 10 min. (A) B. subtilis 168 *amyE*::P_*xyl*_*-comK-*P_*comG*_*-gfp* transformed with DyLight 650-labeled DNA. The labeled DNA (red foci) bound in a DNase I-resistant manner to the competent (green) cells. (B) B. subtilis 168* Δcomk*, which cannot become competent, incubated with fluorescein-DNA. No foci can be seen binding to the cells. (C) B. subtilis grown in LB and incubated with labeled DNA treated with DNase I. No labeled DNA can be seen binding to the noncompetent cells grown in LB. Imaging details are as follows: 100× phase-contrast oil lens; bin, 1×1. (A) Pixel size, 06430 0.06430 0.200; *xy* dimensions, 1,024 by 1,024. Cy5 exposure (exp), 1 s; neutral density (ND), 100%. GFP exp, 0.1 s; ND, 32%. White LED (pol) exp, 0.2 s; ND, 32%. (B) Cy5 exp, 1 s; ND, 100%. Pol exp, 0.2 s; ND, 32%. (C) Size, 480 by 480; fluorescein isothiocyanate (FITC) exp, 1 s; ND, 100%. Pol exp, 0.3 s; ND, 50%.

Under nutrient-limited conditions in the laboratory, only 5 to 50% of the B. subtilis strain 168 population becomes competent. For our microscopy experiments in particular, it is convenient to have a high percentage of competent cells. Therefore, a construct containing exogenously expressed P_*xyl*_-*comK* was used to increase the percentage of competent cells. By growing the cells in competence medium with fructose as a carbon source, repression of the xylose promoter was relieved and the amount of xylose required for the induction of ectopic *comK* was reduced. Under these conditions, the percentage of competent cells increased to approximately 80% of the population. The expression of ectopic *comK* was induced after 4 h of growth, 1 h before high expression of native *comK*. Several nucleotides (nt) were directly incorporated by PCR, i.e., Cy3-dUTP, Cy5-dUTP, and fluorescein-dUTP. A PCR using aminoallyl-dUTP was performed, and the product was subsequently labeled with amine-reactive DyLight 550 or DyLight 650. Alexa Fluor 5-labeled nucleotides were incorporated via the Klenow method. All fluorescent dyes bind covalently to the nucleotides. The ratio of fluorescein-dUTP or aminoallyl-dUTP to dTTP was 1:1. The label incorporation of fluorescein-dUTP, Cy3-dUTP, and Cy5-dUTP, calculated from NanoDrop measurements, was generally between 1.4 and 4.6% for fluorescein-dUTP and between 1.4 and 3% for DyLight 650-dUTP, and on average, label incorporation was 7% for Alexa Fluor 5. The template used was pDG1664, and the erythromycin marker plus the flanking *thrC* regions for integration were amplified from the plasmid. As mentioned above, when B. subtilis successfully takes up DNA, this DNA becomes resistant to DNase I treatment from outside. To exclude unspecific binding of DNA to the cells and to confirm actual uptake, all samples were treated with 10 U of DNase I, as was done in the study of Singl et al. (20), while the incubation time was increased to 10 min at 37°C. The cells were then washed and prepared for microscopy. To confirm that DNA was taken up by competent cells, we incubated B. subtilis 168 *amyE*::P_*xyl*_-*comK-*P_*comG*_*-gfp* with DyLight 650-DNA. The P*_comG_-gfp* construct in this strain is an indicator for competence, with competent cells expressing *gfp* (21). The results shown in Fig. 1A and those in Table S1 in the supplemental material show that labeled DNA bound to competent cells in a DNase I-resistant manner. The results in Fig. 1C show that B. subtilis 168 *amyE*::P*_xyl_-comK* grown in LB medium did not bind labeled DNA, which was expected, as competence is very low when B. subtilis is grown in LB medium. Labeled DNA also was not taken up by a *ΔcomK* strain (Fig. 1B). These results show that labeled DNA associated strongly with competent cells and that the bound DNA was resistant to DNase I, confirming uptake of ssDNA. The same experiment was performed with Streptococcus pneumoniae D39 to see if competent S. pneumoniae was also capable of binding labeled DNA, and indeed, competent S. pneumoniae bound labeled DNA in a DNase I-resistant manner (Fig. S1).

10.1128/mBio.01161-18.1 FIG S1  Competent Streptococcus pneumoniae D39 incubated with DyLight 650-labeled DNA. Labeled DNA bound to competent S. pneumoniae in a DNase I-resistant manner after treatment with 10 U of DNA for 10 min at 37°C. Imaging details are as follows: 100× phase-contrast oil lens; size, 1,024 by 1,024; pixel size, 0.06306 0.06306 0.200; bin, 1×1; Cy5 exp, 8 s; ND, 100%. Pol exp, 0.3 s; ND, 32%. Download FIG S1, TIF file, 0.8 MB. Copyright © 2018 Boonstra et al. 2018 Boonstra et al. This content is distributed under the terms of the Creative Commons Attribution 4.0 International license.

10.1128/mBio.01161-18.5 TABLE S1  DNA binding to cells during competence. Labeled DNA was added in excess to competent cells. After 1 h of incubation, the cells were treated with 10 U of DNA for 10 min at 37°C, washed, and prepared for microscopy. See Fig. 1 in the text. Download TABLE S1, DOCX file, 0.01 MB. Copyright © 2018 Boonstra et al. 2018 Boonstra et al. This content is distributed under the terms of the Creative Commons Attribution 4.0 International license.

### B. subtilis transformed with labeled DNA containing an antibiotic resistance marker formed antibiotic-resistant colonies.

To determine if uptake of labeled DNA would result in antibiotic-resistant transformants, transformation experiments were performed. A negative control was done in which all components were the same, except that no enzyme was added. Amplified PCR or Klenow products and the negative control were incubated for 2 h with DpnI to remove all template DNA. Transformation of the negative control did not yield resistant colonies, confirming that treatment with DpnI removes all template DNA. A positive control using an amplified product with normal deoxynucleoside triphosphates (dNTPs) was also done, to confirm competence of the cells. Transformation with fluorescein-, DyLight 550-, and DyLight 650-labeled DNA resulted in erythromycin-resistant colonies. Transformation with Alexa Fluor 5-DNA did not result in resistant colonies. Transformation with Cy3- or Cy5-DNA was also possible, but despite label incorporation rates similar to those for DyLight-DNA and fluorescein-DNA, only a very small number of resistant colonies were formed. Because transformation with fluorescein-DNA and DyLight-DNA resulted in the highest number of colonies, we compared the transformation efficiencies of fluorescein-dUTP- and DyLight 650-dUTP-labeled DNA with that of unlabeled DNA. The transformation efficiencies of the labeled dyes, with a label incorporation of 3 to 4%, were about 3 times lower for fluorescein-DNA and 2.7 times lower for DyLight 650-DNA than for unlabeled DNA. Due to the relatively high level of label incorporation (30 to 40 labels/1,000 bp), the chance of unlabeled PCR product was very low. It was therefore unlikely that the difference in transformation efficiency was the result of only unlabeled molecules being incorporated. Therefore, to determine whether the integration efficiency was affected by the incorporation of labeled nucleotides, a transformation experiment mixing labeled and unlabeled DNA was performed. The labeled DNA had a high level of label incorporation of 7.4%. The transformation efficiency decreased as the amount of labeled DNA increased (Table 1).

**TABLE 1:** Transformation efficiency^a^

% of labeled DNA used	No. of colonies	No. of colonies transformed/µg DNA
100	57	285
75	136	680
50	236	1,293
25	294	1,611
0	459	3,221

a A total of 375 ng of DNA was added to 150 µl of cells. One hundred microliters of culture was placed on selective LB agar. The percentages of the mixtures of labeled and unlabeled DNA ranged from 100% labeled to 0% labeled. The label incorporation of the labeled DNA was 7.4%.

### Fluorescent DNA localized centrally in the cell.

**FIG 2:**
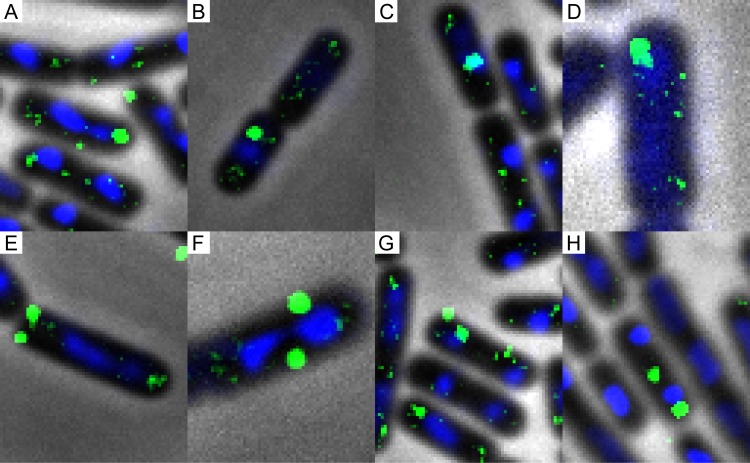
Localization of foci in cells fixed with formaldehyde. The distribution of foci was as follows: at the pole, 34% (A); at the center, 23% (B); at the center overlaying the chromosome, 22% (C); at the pole (partially) overlaying the chromosome, 7% (D); two foci at the pole, 2% (E); two at the center, 3% (F); one focus at the pole and one at the center, 3% (G); and at the division site, 5% (H). The chromosome was stained with DAPI. A total of 3,913 cells were counted, 1,717 of which had DNA foci. Imaging details are as follows: 100× phase-contrast oil lens; bin, 1×1; pixel size, 0.04024 0.04024 0.200; size, 1,024 by 1,024. FITC exp, 0.5 s; ND, 100%. DAPI exp, 0.3 s; ND, 100%. Pol exp, 0.2 s; ND, 32%.

We were interested to see what the localization patterns for labeled DNA would be and whether they would differ from the localization of the competence machinery. In previous studies, 1 to 4 foci of competence proteins per cell were found (1). After 1 h of incubation, we also found 1 to 4 foci per cell for the labeled DNA (Table 2). Although the numbers of foci were similar, the localization of DNA foci did indeed differ from that of the competence machinery. In studies of the localization of the components of the competence machinery, the majority of the foci were localized at the pole, with only 4 to 15% (average, 7.7%), depending on the protein, localized near the center of the cell (1). In cells incubated for 1 h with fluorescein-DNA, treated with DNase I, and fixed with 2% formaldehyde, the labeled DNA was far more often (23%) localized near the center of the cell. In 22% of the cases, the DNA focus overlaid the DAPI (4′,6-diamidino-2-phenylindole)-stained chromosome, although with this imaging technique, direct interaction with the chromosome cannot be accurately determined. Single foci localized at an average of 43% in the center of the cell and 39% at the pole after 1 h of incubation. Figure 2 shows the localization of fluorescein-DNA. The higher percentages of localization near the center of the cell and colocalization with the chromosome of labeled DNA were in accordance with our previous observation that the labeled DNA was internalized.

**TABLE 2:** Percentages of cells with certain numbers of DNA foci

No. of foci	No. (%) of cells with indicated no. of foci (*n* = 599 cells counted)
1	465 (77.6)
2	110 (18.4)
3	22 (3.7)
4	2 (0.3)

### Visualization of the uptake of multiple DNA molecules.

**FIG 3:**
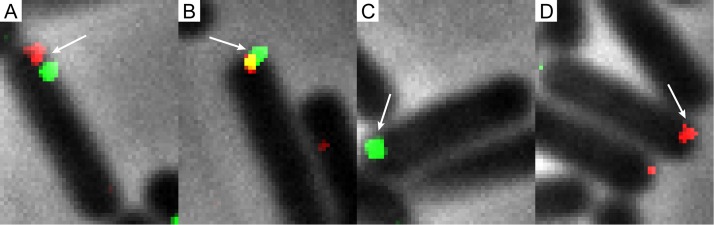
Colocalization of DyLight 650 DNA with fluorescein-labeled DNA. The distribution of (co)localization was as follows: fluorescein-DNA and DyLight 650-DNA localizing in 1 cell, 17.2% (103 cells) (A); overlapping fluorescein-DNA and DyLight 650-DNA, 8.8% (53 cells) (B); only fluorescein-DNA, 43.2% (259 cells) (C); and only DyLight 650-DNA, 31% (184 cells) (D). A total of 599 cells were counted. Imaging details are as follows: 100× phase-contrast oil lens; bin, 1×1; size, 896 by 896; pixel size, 0.06455 0.06455 0.050. FITC exp, 0.5 s; ND, 100%. Cy5 exp, 2 s; ND, 100%. Pol exp, 0.15 s; ND, 32%.

Previous research has shown that competent B. subtilis cells can take up multiple DNA molecules, with an average of 20 to 53 uptake sites per cell (22), and bind 30 to 45 DNA molecules (10 to 15 after 1 min of exposure to transforming DNA) (23). It has also been shown that competent cells primarily contain only 1 competence machinery and that when 2 or more are present, likely only one is active upon the addition of DNA, because RecA localizes at one of the competence machineries (1, 2, 18). It is not known how many ComEC channels are present in one competence machinery. In our results, the majority of cells contained a single DNA focus. However, it was not clear whether single foci contained multiple DNA molecules. To obtain information about the number of DNA molecules in single foci, DyLight 650-DNA and fluorescein-DNA were mixed in equal amounts and competent cells were incubated with the mixture of labeled DNA. The two labeled dyes have similar transformation efficiencies. After 1 h, colocalization of the dyes was present in 9% of the cells containing foci (Fig. 3). In 17% of the cells, the foci were located close to each other inside the cell but did not overlap. The occurrence of internalization of two different colors of labeled DNA thus lay at 26%. Forty-three percent of the cells only had fluorescein foci, and 31% only had DyLight-650 foci. Although actual full colocalization of two different DNAs only occurred in 9% of the cases, some of the foci still might have contained multiple DNA molecules of the same color. It is therefore likely that 9% was somewhat of an underestimation. This, combined with the facts that there were 17% of cells with localization of two colors of DNA and that 22% of the cells had two or more foci when transformed with a single color of DNA, shows for the first time microscopically that B. subtilis can take up multiple DNA molecules in a parallel fashion.

### **Labeled DNA colocalized with ComFC, RecA**,** and a homologous locus on the chromosome.**

**FIG 4:**
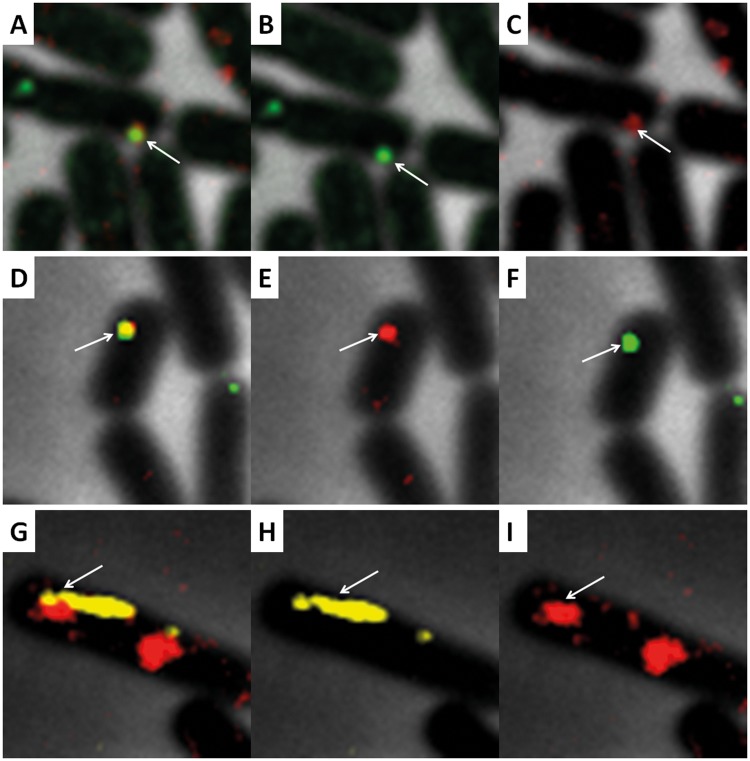
Colocalization of DyLight 650-labeled DNA with ComFC-GFP. Samples were incubated for 10 min with labeled DNA. (A) Overlay of ComFC-GFP and DyLight 650-DNA. (B) ComFC-GFP. (C) DyLight 650-DNA. Among cells containing both DNA and ComFC foci, colocalization occurred in 23%. (D to F) Colocalization of ParB-mKate with fluorescein-labeled DNA. (D) Overlay of ParB-mKate and Fluorescein DNA. (E) ParB-mKate. (F) Fluorescein-DNA. Colocalization occurred at 3% after 10 min and 7% after 1 h. (G to I) Colocalization of the filamentous form of RecA-YFP with DyLight 650-labeled DNA. (G) Overlay of RecA-YFP and DyLight 650-DNA. (H) RecA-YFP. (I) DyLight 650-DNA. Of cells containing both DNA and RecA foci, colocalization was at 26% after 15 min and 15% after 1 h of incubation. Imaging details are as follows: 100× phase-contrast oil lens; size, 640 by 640; pixel size, 0.06430 0.06430 0.200; bin, 1×1. (A to C) GFP exp, 0.8 s; ND, 100%. Cy5 exp, 2 s; ND, 100%. Pol exp, 0.25 s; ND, 32%. Cy5 exp, 2 s; ND, 100%. Pol exp, 0.25 s; ND, 32%. (D to F) mCherry exp, 0.8 s; ND, 100%. FITC exp, 2 s; ND, 100%. Pol exp, 0.2; ND, 32%. (G to I) YFP exp, 1 s; ND, 32%. Cy5 exp, 0.8 s; ND, 100%. Pol exp, 0.25 s; ND, 32%.

After uptake of the labeled DNA was confirmed, we wanted to know whether labeled DNA colocalized with a competence and a recombination protein. Therefore, colocalization with ComFC-green fluorescent protein (GFP) and RecA-yellow fluorescent protein (YFP) was determined. ComFC is a component of the competence machinery with an as-yet-unknown function, but it has been shown to interact with the ATPase ComFA (24). The number of ComFC foci and the localization (Fig. S2) were similar to those found by Kaufenstein et al. (1). The constructs formed resistant colonies after transformation. To determine colocalization with ComFC, the cells were incubated for 10 min with labeled DNA and subsequently fixed with 2% formaldehyde. The cells were then washed and treated with DNase I as described above. We found that 23% colocalization of DNA foci with ComFC foci occurred (Fig. 4A to C and Table 3). As the labeled DNA successfully colocalized with the competence machinery, we were interested in whether colocalization with the recombination protein RecA could also be observed. For this experiment, the BD4477 strain was used, which contains genes expressing a RecA-YFP fusion (2). Of the cells showing both RecA-YFP and DyLight 650 foci, colocalization occurred in 26% of the cells at 15 min and in 15% after 1 h of incubation (Table 4). RecA is the main protein responsible for homologous recombination (15–17). During transformation, Kidane and Graumann saw filamentous RecA being formed upon the addition of exogenous unlabeled DNA (18). They proposed that this filamentous form is likely the form RecA takes when actively searching for homologous regions. We also observed the filamentous form of RecA, and this form could be seen to colocalize with the labeled DNA (Fig. 4G to I). We were curious as to whether we could see the labeled DNA colocalize at a specific locus on the chromosome, as this would more accurately show interaction with the chromosome. Because labeled DNA capable of integrating into the *thrC* locus on the chromosome was used, it should be possible to see the labeled DNA colocalizing with this locus. A *parB-gfp* or *parB-mkate* fusion construct with a single *parS* site within the *parB* gene was cloned into the *thrC* locus of B. subtilis. The *parB/parS* gene originates from Lactococcus lactis plasmid pLP712 (25). After 10 min of incubation with DNA and subsequent fixing with 2% formaldehyde and treatment with DNase I, fluorescein-DNA colocalized with ParB-mKate at a rate of 4% in cells containing both ParB-mKate and fluorescein foci. After 1 h of incubation, 9% colocalization could be seen (Fig. 4D to F and Table 5). Similar colocalization percentages could be obtained for labeled DyLight 650-DNA and ParB-GFP, which showed 3% colocalization at 10 min and 7% colocalization after 1 h of incubation.

10.1128/mBio.01161-18.2 FIG S2  Localization of ComFC-GFP foci in B. subtilis 168 *amyE*::*P_xyl_-comK_comFC-gfp*. Of the 1,571 cells counted, 34.5% had 1 focus, 18.6% 2 foci, 2.5% 3 foci, and 0.4% 4 foci. The foci were localized 70% at the pole and 30% at the division site/center of the cell. Imaging details are as follows: 100× phase-contrast oil lens; *xy* dimensions, 1,024 by 1,024; pixel size, 0.06455 0.06455 0.200; bin, 1×1. GFP exp, 0.8 s; ND, 100%. Pol exp, 0.05 s; ND, 32%. Download FIG S2, TIF file, 0.3 MB. Copyright © 2018 Boonstra et al. 2018 Boonstra et al. This content is distributed under the terms of the Creative Commons Attribution 4.0 International license.

**TABLE 3:** Colocalization of DyLight 650-labeled DNA and ComFC-GFP

Type of foci	No. (%) of cells with indicated type of foci (*n* = 3,369 cells counted)	% colocalization of DNA-ComFC^a^
DNA	1,104 (33)	
ComFC	872 (26)	
Colocalized	197 (6)	23

a The percentage of colocalization of labeled DNA and ComFC was calculated by dividing the total number of cells with colocalizing foci by the total number of cells with ComFC foci.

**TABLE 4:** Colocalization of DyLight 650-labeled DNA with RecA-YFP after different times of incubation with DNA

Incubation time (no. of cells counted)	Type of foci	No. (%) of cells with indicated type of foci	% colocalization of DNA-RecA^a^
15 min (1,749)	DNA	890 (51)	
	RecA-YFP	115 (7)	
	Colocalized	30 (2)	26

1 h (4,538)	DNA	1,992 (44)	
	RecA-YFP	631 (14)	
	Colocalized	98 (2)	15

a The percentage of colocalization of labeled DNA and RecA-YFP was calculated by dividing the total number of cells with colocalizing foci by the total number of cells with RecA-YFP foci.

**TABLE 5:** Colocalization of DNA and ParB-mKate after 10 min and 1 h of incubation

Incubation time (no. of cells counted)	Type of foci	No. (%) of cells with indicated type of foci	% colocalization of DNA-ParB^a^
10 min (1,292)	DNA	310 (36)	
	ParB-mKate	470 (24)	
	Colocalized	36 (1.5)	4

1 h (2,200)	DNA	1,205 (55)	
	ParB-mKate	1,173 (53)	
	Colocalized	107 (5)	9

a The percentage of colocalization of DNA and ParB-mKate was calculated by dividing the total number of cells with colocalizing foci by the total number of cells with ParB-mKate foci.

### B. subtilis was easily transformed within a microfluidics setting.

**FIG 5:**
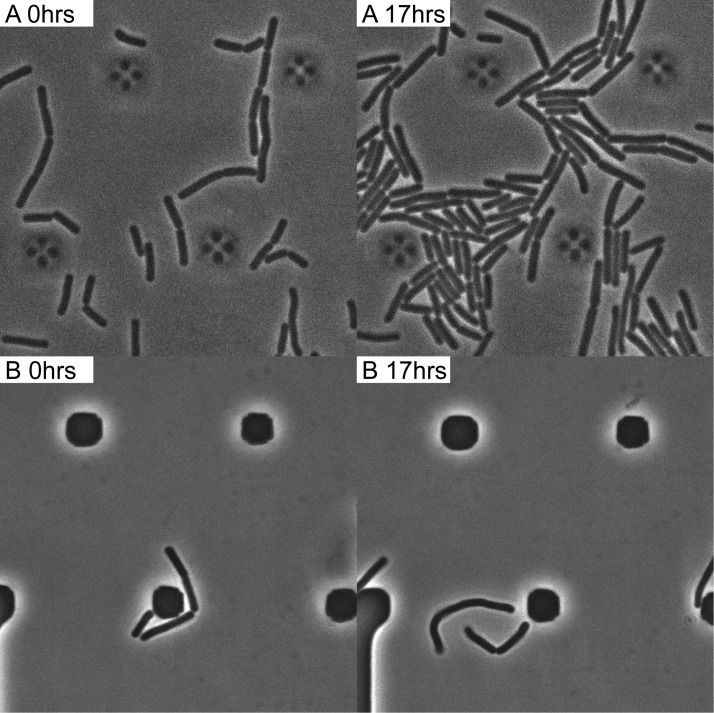
B. subtilis 168 *amyE*::P*_xyl_-comK* transformed with DyLight 650-labeled *thrC*::P_*spank*_*-gfp*(ery) DNA. (A) Phase-contrast image of cells grown in the presence of erythromycin (growth rate = 0.51), confirming successful transformation and expression of the erythromycin cassette. (B) B. subtilis 168 *amyE*::P*_xyl_-comK*. Control where no DNA is added. Cells show no or reduced growth (growth rate = 0.046) in the presence of erythromycin. Imaging details are as follows: 100× phase-contrast oil lens; size, 640 by 640; pixel size, 0.06430 0.06430 0.200. Pol exp, 0.2 s; ND, 32%.

Time-lapse microscopy experiments are often done on solid medium. However, for transformation experiments, a liquid medium is much more convenient. A microfluidics system was therefore used for further experiments. The fact that the medium actively flows through the system allows rapid changing of conditions, which is a great advantage of microfluidics over time-lapse microscopy on solid-medium slides. First, it was determined whether transformation with labeled DNA occurred in the microfluidics setup. B. subtilis 168 P*_xyl_-comK* was incubated with DyLight 650-labeled *thrC* P_*spank*_*-gfp*(ery). Using a *gfp* construct allowed for determination of the time needed before gene expression occurred after uptake of DNA and whether cell division occurred before gene expression. At all stages of the experiment, 1 mM IPTG (isopropyl-β-d-thiogalactopyranoside) was present in order to induce the expression of P*_hyspank_-gfp*. Cells were incubated with DNA that was added to supernatant from the culture for 2 h. The supernatant contained competence signaling molecules, which may enhance maintenance of the competence state. The *comK* inducer xylose was present at this stage of the experiment, but not the subsequent steps. After 2 h, fresh medium with DNase I was added for 10 min. Fresh medium without DNase I and antibiotics was added for 1 h to stimulate exit from the competence state. Fresh medium containing antibiotics was added after 3 h 10 min, and the experiment was run for a further 16 h. Although only a few cells clearly showed *gfp* expression above the background level of *Bacillus* cells, the cells grew in the presence of erythromycin, with a growth rate of 0.51 (determined by measuring the cell length with the measuring tool in ImageJ and calculating the slope by linear regression) (Fig. 5A; Movie S1). A control with no DNA added showed no growth or a reduced growth rate of 0.046 after the addition of antibiotics (Fig. 5B; Movie S2).

10.1128/mBio.01161-18.6 MOVIE S1  Transformation of B. subtilis 168 with DyLight 650 *thrC*::P*_hyspank_-gfp* DNA. Very few cells showed expression of *gfp* above the background level of B. subtilis. Imaging details are as follows: size, 640 by 640; pixel size, 0.06430 0.06430 0.200; bin, 1×1. GFP exp, 0.8 s; ND, 32%. Pol exp, 0.2; ND, 32%. Download MOVIE S1, MOV file, 13 MB. Copyright © 2018 Boonstra et al. 2018 Boonstra et al. This content is distributed under the terms of the Creative Commons Attribution 4.0 International license.

10.1128/mBio.01161-18.7 MOVIE S2  B. subtilis 168 grown in microfluidics system with no DNA added. Antibiotics were added after 3 h 10 min. Imaging details are as follows: 100× phase-contrast oil lens; size, 640 by 640; pixel size, 0.06430 0.06430 0.200; bin, 1×1. Pol exp, 0.2; ND, 32%. Download MOVIE S2, AVI file, 4.1 MB. Copyright © 2018 Boonstra et al. 2018 Boonstra et al. This content is distributed under the terms of the Creative Commons Attribution 4.0 International license.

### Expression of integrated DNA occurred before cell division.

**FIG 6:**
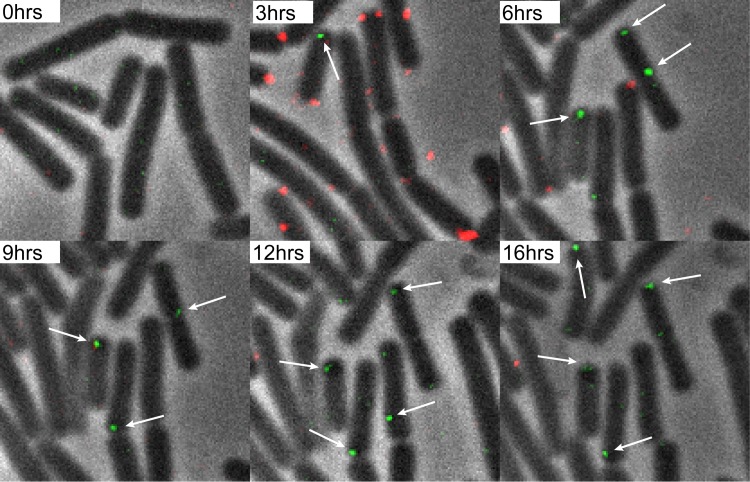
Transformation of B. subtilis 168 *amyE*::P*_xyl_-comK* with DyLight 650-labeled *thrC*::P*_spank_-parB-gfp* DNA. On average, foci became visible after 6 h and 45 min. Fifteen percent of the cells expressed *gfp* or produced daughter cells expressing *gfp*. The arrows indicate a selection of foci within cells expressing *gfp*. Imaging occurred every 15 min. Imaging details are as follows: 100× phase-contrast oil lens; size, 640 by 640; pixel size, 0.06430 0.06430 0.200; bin, 1×1; Cy5 exp, 0.8 s; ND, 32%. GFP exp, 0.8 s; ND, 32%.

Next, we wanted to answer the question of whether expression from integrated exogenous labeled DNA could be visualized. During competence, cell division is halted, and it would be interesting to know if expression of the integrated DNA coincides with the resumption of cell division. To overcome the problem posed by the low expression of *gfp* and the background fluorescence of B. subtilis cells, we switched to using a DyLight 650-labeled *thrC* P_*spank*_*-parB-gfp*(ery) construct for transformation. The formation of foci by this construct allows for better distinction between background fluorescence and the expression of *gfp* from the integrated exogenous DNA. The timing of the changing of the medium was the same as described in the previous section. When transformed with labeled *thrC* P_*spank*_*-parB-gfp*(ery), cells could be seen to take up labeled DNA and divide in the presence of erythromycin (Fig. 6). After the addition of antibiotics, a total of 31% of the cells continued to divide, while the average doubling time was 61 min. As expected, it was then possible to distinguish between background fluorescence and expression from the integrated DNA, as foci first started to become visible 1 h 30 min after the addition of DNA. We found that, on average, foci became visible 6 h 45 min after the addition of DNA and 4 h 45 min after the addition of fresh medium. Not all of the cells that were dividing after the addition of antibiotics expressed *gfp*, and not all of the cells expressing *gfp* divided after addition of antibiotics. In total, 15% of the cells expressed *gfp* or produced daughter cells expressing *gfp* (Fig. 6; Movie S3). Notably, under these conditions, cell division was not required for the expression of GFP and 49% of the *gfp*-expressing cells did not divide before *gfp* was expressed.

10.1128/mBio.01161-18.8 MOVIE S3  B. subtilis 168 transformed with Dylight 650-labeled *thrC_*P*spank-parB-gfp*(*ery*). Cells not only divided in the presence of antibiotics but also expressed *parB-gfp*. Imaging details are as follows: 100× phase-contrast oil lens; size, 640 by 640; pixel size, 0.06430 0.06430 0.200; bin, 1×1. Cy5 exp, 0.8 s; ND, 32%. GFP exp, 0.8 s; ND, 32%. Pol exp, 0.2 s; ND, 32%. Download MOVIE S3, AVI file, 1.8 MB. Copyright © 2018 Boonstra et al. 2018 Boonstra et al. This content is distributed under the terms of the Creative Commons Attribution 4.0 International license.

### Replacement of a homologous locus by exogenous DNA could be visualized.

Despite the increase in localization of labeled DNA with the *thrC* locus increasing over time, the total occurrence of colocalization was low compared to the colocalization with ComFC and RecA. We therefore determined whether the replacement of this locus on the chromosome can be visualized, as this would definitively show that the exogenous DNA integrated in this locus. First, a time-lapse microscopy experiment with unlabeled DNA on polyacrylamide slides containing medium and 1 mM of IPTG was performed. B. subtilis 168 *amyE*::P*_xyl_-comK thrC*::P_*spank*_-*parB-mkate* was transformed with pDG1664. The *parB-mkate* construct in the *thrC* locus should disappear when it is replaced by homologous exogenous DNA. Samples were imaged every 10 min for a total time of 4 h. A control containing no DNA was done to ensure that loss of foci was indeed the result of integration of transformed DNA. Ninety minutes after the addition of DNA and 60 min after the start of the time-lapse experiment, foci started disappearing in the samples incubated with DNA. In the control with no added DNA, the foci were still visible after 200 min of imaging, confirming that the disappearance of foci was due to the integration of DNA (Fig. S3). To exclude the possibility of chromosomal reorganization during transformation causing the disappearance of foci, the *amyE*::P_*xyl*_-*comK-thrC*::*parB-gfp* strain was incubated with DNA integrating in the *sacA* locus. After 4 h, 94% of the cells still contained clear foci, showing that the disappearance of foci in the previous experiment was indeed the result of replacement of the *thrC* locus (Fig. S4). To determine if homologous labeled DNA also replaces the *parB-gfp/parS*, we switched to a microfluidics system, with the same timing of the changing of conditions described previously. Images were taken every minute to determine colocalization and every 15 min to determine focus displacement. We transformed B. subtilis 168 *amyE*::P*_xyl_-comK thrC*::P_*spank*_*-parB-gfp*(ery) with DyLight 650-labeled *thrC-spec* DNA (from pDG1731) or B. subtilis 168 *amyE*::P*_xyl_-comK thrC*::P_*spank*_*-parB-mkate*(ery) with fluorescein-labeled *thrC-spec* DNA. Transforming with homologous DNA containing a spectinomycin cassette allowed us to also select for successful transformation, in addition to the disappearance of foci. When imaging every minute, significant bleaching of the dye does occur between 10 and 20 excitations, which made the determination of colocalization difficult. Another factor making capture of colocalization more difficult is the fact that the chromosome is not fixed in one place, but moves around the cell (Movie S4). Although with the current setup, we were not able to clearly determine colocalization of a specific locus on the chromosome with labeled DNA, replacement of the homologous region by labeled exogenous DNA was observed (Movie S5). As for the unlabeled DNA, the disappearance of foci became visible approximately 90 min after the addition of DNA. For the *parB-mkate* strain, the percentage of cells with no foci increased from 34% at 30 min to 51% after 4.5 h (Table 6; Movie S5). For the *parB-gfp* strain, the percentage of cells with no foci increased from 9% to 41% after the addition of DNA (Table 6). The percentage of cells containing 2 foci was higher after 4.5 h of growth. This likely was the result of the resumption of cell division, as *Bacillus* bacteria are capable of initiating multiple rounds of replication at once. The inability to clearly distinguish a septum likely also played a role, as it could lead to a false focus count. As the addition of antibiotics occurred after 3.1 h and because it might have taken some time for the concentration of antibiotics to reach selective concentrations, it is possible that cells that did not express the resistance cassette could have grown for a while after the addition of antibiotics. The transformed cells grew in the presence of spectinomycin after longer exposure to selective medium, confirming successful transformation (Movie S5). Although cells grew in the presence of spectinomycin, in some cells, ParB-mKate foci remained visible for several hours.

10.1128/mBio.01161-18.3 FIG S3  (A) Transformation of B. subtilis 168 *amyE*::P*_xyl_-comK_thrC*::*parB-mkate2* with pDG1664. The ParB-mKate foci disappear as the *parB-mkate* construct is replaced by pDG1664. (B) B. subtilis 168 *amyE*::P*_xyl_-comK_thrC*::*parB-mkate2* without exogenous DNA. The control was performed under the same conditions as the transformed sample, but no DNA was added. The foci are still present after 200 min, confirming that ParB-mKate remains bound to the *parS* site in the absence of homologous exogenous DNA. Imaging details are as follows: 100× phase-contrast oil lens; size, 1,024 by 1,024; pixel size, 0.06430 0.06430 0.200; bin, 1×1. mKate exp, 1 s; ND, 50%. Pol exp, 0.2 s; ND, 32%. Download FIG S3, TIF file, 1 MB. Copyright © 2018 Boonstra et al. 2018 Boonstra et al. This content is distributed under the terms of the Creative Commons Attribution 4.0 International license.

10.1128/mBio.01161-18.4 FIG S4  B. subtilis 168 *thrC_*P*_spank_-parB-gfp* transformed with Psac-km plasmid. Image taken after 4 h of incubation with plasmid that integrates into the *sacA* locus, showing that disappearance of ParB foci is not the result of chromosomal reorganization. Imaging details are as follows: 100× phase-contrast oil lens; size, 512 by 512; pixel size, 0.06430 0.06430 0.200; bin, 1×1. GFP exp, 0.5 s; ND, 32%. Pol exp, 0.3 s; ND, 50%. Download FIG S4, TIF file, 0.8 MB. Copyright © 2018 Boonstra et al. 2018 Boonstra et al. This content is distributed under the terms of the Creative Commons Attribution 4.0 International license.

10.1128/mBio.01161-18.9 MOVIE S4  Transformation of B. subtilis 168 *amyE*::P*_xyl_-comK_thrC*::P_*spank*_*-parB-gfp*(ery) with DyLight 650-labeled *thrC-spec* DNA. Imaging was done every minute for 15 min. The chromosome is not fixed in one place during competence; this combined with bleaching of fluorescein after 15 excitations makes determination of colocalization difficult. Imaging details are as follows: 100× phase-contrast oil lens; size, 640 by 640; pixel size, 0.06430 0.06430 0.200; bin, 1×1; Cy5 exp, 0.8 s; ND, 32%. GFP exp, 0.8 s; ND, 32%. Pol exp, 0.2 s; ND, 32%. Download MOVIE S4, MOV file, 1 MB. Copyright © 2018 Boonstra et al. 2018 Boonstra et al. This content is distributed under the terms of the Creative Commons Attribution 4.0 International license.

10.1128/mBio.01161-18.10 MOVIE S5  Transformation of *B. subtilis* 168 *amyE*::P_*xyl*_*-comK*_*thrC*::P_*spank*_*-parB-gfp*(ery) with DyLight 650-labeled *thrC-spec* DNA. Imaging was done every 15 minutes. We were unable to determine colocalization when imaging every 15 min; however, disappearance of ParB-mkate foci was visible. Disappearance of foci became visible again approximately 90 min after the addition of DNA. The percentage of cells with no foci increased from 34% at 30 min after the addition of DNA to 51% after 4.5 h. Imaging details are as follows: 100× phase-contrast oil lens; size, 640 by 640; pixel size, 0.06430 0.06430 0.200; bin, 1×1. FITC exp, 0.5  s; ND, 32%. mCherry exp, 1 s; ND, 32%. Pol exp, 0.3 s; ND, 32%. Download MOVIE S5, AVI file, 5.6 MB. Copyright © 2018 Boonstra et al. 2018 Boonstra et al. This content is distributed under the terms of the Creative Commons Attribution 4.0 International license.

**TABLE 6:** Disappearance of ParB-mKate and ParB-GFP foci after addition of labeled DNA

Time point	Value for cells with indicated type and number of foci^a^							
	ParB-mKate				ParB-GFP			
	Total no. of cells counted	% of cells with:			Total no. of cells counted	% of cells with:		
		1 focus	2 foci	No foci		1 focus	2 foci	No foci
30 min	211	53	13	34	211	72	20	9
1.5 h	375	44	10	47	217	62	24	14
2.5 h	483	39	10	52	282	69	9	22
3.5 h	669	33	14	53	421	44	27	44
4.5 h	729	27	22	51	547	23	36	41

a The number of foci were counted after the addition of labeled DNA capable of integration in the *thrC* locus. The number of cells without foci increased over time.

## DISCUSSION

We set out to answer several questions. Can B. subtilis take up fluorescently labeled DNA via transformation and does this result in resistant transformants? Can the labeled DNA be seen to colocalize with the competence machinery, RecA, and the chromosome? What is the timing of the transformation process with regard to the integration and expression of exogenous DNA? And finally, does the expression of exogenous DNA coincide with resumed cell division? First, various covalently labeled fluorescent DNA species were tested in order to develop a method to answer these questions. It was found that of the DNA labeling methods tested, fluorescein-DNA and DyLight 650-DNA are successfully taken up by competent B. subtilis at a relatively high efficiency. Furthermore, labeled DNA binds to competent but not to noncompetent B. subtilis in a DNase I-resistant manner, confirming the specificity of binding to competent cells and the uptake of DNA (Fig. 1). The labeled DNA binds to competent cells in a DNase I-resistant manner not only for B. subtilis but also for competent Streptococcus pneumoniae. The transformation efficiency is, however, lower when B. subtilis cells are transformed with labeled DNA (Table 1). Labeled nucleotides are incorporated into both strands over the entire length of the DNA; however, there is a possibility that stretches of the DNA (e.g., 30 nt long, in view of the 3 to 4% incorporation of label) do not contain labeled nucleotides. The lower transformation efficiency can mean that labeled DNA integrates less efficiently or that only those parts of the DNA molecule that do not contain labeled nucleotides integrate, taking along the labeled residues in between the two integration sites. It is also possible that the presence of foreign nucleotides in exogenous DNA results in mutations. Another possibility is that the transcription complex has difficulties with the presence of foreign nucleotides, resulting in reduced rates of transcription. The lower transformation efficiency with labeled DNA may also indicate that some components of the recombination machinery have problems with the presence of labeled nucleotides. Our success with fluorescein and DyLight 650 likely lies in the physical properties of these dyes. Stingl et al. fluorescently labeled DNA using Cy3, but this labeling method did not result in uptake of labeled DNA by B. subtilis (20). We also attempted transformation with Cy3- and Cy5-labeled DNA, using a different labeling method, but even with the hypercompetent strain, only a very few resistant colonies were formed. DyLight 650 and DyLight 550 are negatively charged and have a higher solubility in water than Cy5 and Cy3. During transformation, negatively charged DNA is transported through the water-filled ComEC channel. Therefore, the charge and solubility of the dye, along with its size, likely are important factors in the ability of the competence machinery to take up modified DNA. Research by Berlatzky et al., who labeled the entire chromosome with fluorescent nucleotides, shows that replication occurs when cells are depleted of natural nucleotides and nonnatural nucleotides are incorporated instead (26). B. subtilis is thus capable of utilizing nonnatural nucleotides for multiple processes.

When competent B. subtilis is transformed with both DyLight 650-DNA and fluorescein-DNA (Fig. 3), full colocalization of the two is seen in 9% of the cells, and in 17%, both red and green foci are found in the cell. The majority of the cells have either DyLight 650 foci (31%) or fluorescein foci (43%); however, it cannot be excluded that some of the foci contain multiple DNA molecules of one type of labeled DNA. The results show microscopically that B. subtilis can take up multiple DNA molecules, likely simultaneously. Although only 1 to 4 competence machinery foci are found (1), previous research using radioactively labeled DNA led to a calculation of 20 to 53 DNA uptake sites per cell (22), or 10 to 15 DNA molecules bound at a given time (23). The use of fusions of superresolution-capable fluorescent proteins to ComEC could definitively determine the number of ComEC channels in the competence machinery and, thus, the number of uptake sites. Many fluorescent dyes can also be used for superresolution microscopy (27), which further opens up exciting possibilities, such as an accurate determination of the number of DNA molecules transported during competence.

After confirmation of uptake, we also looked at colocalization of labeled DNA with ComFC, RecA, and the chromosome. The localization of the labeled DNA differs from that of the components of the competence machinery, with a much higher number of cells with foci localized centrally in the cells being found for DNA than for the competence proteins, further confirming successful internalization (Fig. 2). The labeled DNA colocalizes, on average, at a rate of 22% with the chromosome. Although labeled DNA is more often localized at the center of the cell, the labeled DNA can be seen to colocalize with ComFC, with 23% colocalization after 10 min (Fig. 4A to C, Table 3). Labeled DNA also colocalizes with RecA during transformation, with 26% colocalization after 15 min and 15% colocalization after 1 h (Fig. 4G to I, Table 4). Formation of the actively searching filamentous form of RecA (18) in the presence of labeled DNA is also observed. The colocalization with specific sites on the chromosome, however, is lower than colocalization with the chromosome and ComFC and RecA. After 10 min of incubation, only 4% colocalization with the *thrC* locus is found. When cultures are incubated with the labeled DNA for 1 h, the percentage of colocalization is slightly higher, i.e., 9% (Fig. 4D to F, Table 5). The relatively low level of colocalization could be a result of the searching-for-homology process, as colocalization does increase over time. However, the lower transformation efficiency may also mean that the unlabeled regions of the exogenous labeled DNA are primarily used for homologous recombination into the chromosome.

After uptake of labeled DNA and colocalization of labeled DNA with ComFC and with RecA was confirmed, we set out to investigate the timing of the transformation process. We found that hypercompetent B. subtilis can easily be transformed in a microfluidics system and that cells to which no DNA is added do not grow in the presence of antibiotics. One of the questions we wanted answered was, how long does it take before imported DNA is integrated into the chromosome? We found that in both the solid-medium time-lapse microscopy experiment and the microfluidics setup, foci start disappearing approximately 90 min after the addition of DNA (Table 6; Fig. S3 and Movie S5 in the supplemental material), with the percentage of cells with no foci increasing from 34% to 51% after 4.5 h for the *parB-mkate* strain and from 9% to 41% for the *parB-gfp* strain (Table 6). The disappearance of the ParB-GFP/ParB-mKate foci confirms that the exogenous DNA is integrated into the homologous region on the chromosome. Ninety minutes before the disappearance of foci is quite a long time, especially when taking into account that DNA is taken up rapidly, with a speed of 80 bp/s (28). This means that our construct should be fully transported after approximately 31 s. Due to technical limitations, such as bleaching of the dye, we were not able to clearly visualize colocalization with a specific locus. Because of this, we could not accurately determine how long it takes for the recombination system to find the homologous region and begin strand invasion. A possible reason for the long time before foci disappear is that the ParB in our construct is able to bind single-stranded DNA during recombination and, therefore, is not completely displaced during recombination. The *Bacillus* ParB (Spo0J) is able to bind single-stranded DNA (29–32), and it is therefore likely that the ParB from the L. lactis plasmid in our construct is able to do so as well. Also, initially, two different strands are present: one with the integrated DNA and one with the original *parB/parS* construct. The use of a nonreplication-associated DNA-binding protein may solve this problem. If only the unlabeled parts of the DNA molecule are involved in the recombination events, this may also result in situations where parts of the original construct remain. The use of a more photostable dye should solve the technical problems with determining colocalization with the homologous region and could definitively determine whether the labeled nucleotides are also integrated.

We also wanted to know how long it takes before expression of the exogenous DNA occurs. In this setup, it also takes at least 90 min before the expression of *gfp* from the integrated DNA first becomes visible. The average time for the expression of GFP is 6 h 45 min after the addition of DNA and 4 h 45 min after the addition of fresh medium, with an average doubling time of 61 min (Fig. 6; Movie S3), which is approximately double that of division in a shake flask. Haijema et al. found that competent cells resumed replication and growth 2 to 3 h after dilution in fresh medium (33). At an average of 4 h 45 min before the expression of *gfp* from the integrated DNA, expression likely coincides with the cell exiting from the competent state, as RNA production is reduced during competence (34). It should be noted that the experiments were performed using an artificial system fto induce competence (described in detail in Hahn et al. [35]). We induced ectopic *comK* 1 h before entry into natural competence in order to increase the number of cells entering competence. Ectopically induced *comK* bypasses regulation by AbrB, DegU, and SinR and remains subject to degradation by the MecA and ClpC/-P degradation complex (35). Our results indicate that exit from the competent state is not strongly affected once the inducer is removed, as both the doubling time and timing of exit are approximately twice the doubling time and timing of exit from a competence state in a shake flask. The transformation efficiency in the microfluidics system is relatively high, as 30% of the cells are able to divide in the presence of the selective antibiotics. The expression of *gfp* originating from the exogenous DNA, however, is lower, with 15% of the cells expressing *gfp*. Multiple factors may play a role in the lower expression of *gfp* than of the resistance gene. The resistance gene is expressed from a constitutive promoter, whereas the *gfp* gene is under the control of an inducible P_*hyspank*_ promoter. Alternatively, the oxygen concentrations within the microfluidics system could be too low in some cells. A possible explanation for the fact that not all cells expressing *gfp* are dividing and *vice versa* is that only the portions of DNA which do not contain labeled nucleotides can recombine.

The final question we wanted answered was, does the expression of integrated DNA coincide with the resumption of cell division? Halting of cell division and replication gives the bacteria time to integrate exogenous DNA before resuming growth. It is exciting that under these conditions, expression occurs before the resumption of cell division. It was found that 49% of the cells express *gfp* before division. It is most probable that the relatively long average time before the expression of integrated DNA (4 h 45 min after the addition of fresh medium) is primarily the result of the recombination and repair process. Replication might also be needed for the expression of exogenous DNA, as the mismatch repair proteins MutS and MutL localize at mismatches that emerge from DNA polymerase (36). It has also been found that mutations in *mutSL* of S. pneumoniae reduce transformation efficiency when exogenous DNA has approximately 5% difference from the sequence of the recipient (37). It is therefore likely that for successful expression, mismatches need to be repaired. Expression thus likely coincides with resumed replication but can occur before full exit from the competent state. The expression of exogenous DNA before division may be beneficial, as many antibiotics are capable of inhibiting cell division or killing cells during division by inhibiting cell wall synthesis. More generally, if transformation is primarily a method of obtaining genes that improve fitness, being able to express newly acquired genes (possibly providing a fitness benefit) before division makes sense, as division is an energy-intensive process.

To summarize, we show that B. subtilis can take up DyLight 650- and fluorescein-labeled DNA. Labeled DNA is taken up at the pole, as it can be seen to colocalize with ComFC of the competence machinery. The labeled DNA colocalizes with the (actively searching) main recombination protein RecA. Moreover, transformation with exogenous DNA in a microfluidics system results in the replacement of a homologous locus on the chromosome. The transformation efficiency in the microfluidics system is high, and we confirmed expression of the exogenous DNA. Interestingly, we found that the expression of DNA can occur before cell division. Because the competence machinery is conserved among naturally competent species, the use of labeled DNA is broadly applicable in the study of DNA uptake. It provides a powerful method for the detailed investigation of all processes during transformation. Furthermore, it may be used to study other types of horizontal gene transfer, such as phage transduction or conjugation.

## MATERIALS AND METHODS

### Strain construction.

The B. subtilis 168 *amyE*::P*_xyl_-comK-cm comFC-gfp-tet* strain was obtained by USER cloning. The fusion construct comprises *gfp-DSM* (developed by Koninklijke DSM N.V.) with a flexible linker from JWV500 (38), made using primers prMB94 and prMB62, and then the C terminus of *comFC* with primers prMB97 and prMB89, followed by the pBEST309 tetracycline region (39) using primers prMB93 and prMB100 and the upstream flanking region of *comFC* with prMB88 and prMB62. The different components of the construct were obtained by PCR with PfuX7 (40), treated with USER enzyme (NEB), ligated overnight at 4°C, and transformed directly into B. subtilis 168 *amyE*::P*_xyl_-comK-cm* for integration into the native locus. The strain was checked for proper integration by PCR and sequenced. B. subtilis 168 *amyE*::P*_xyl_-comK thrC*::P_*spank*_-*parB-gfp* and B. subtilis* 168 amyE*::P*_xyl_-comK thrC*::P*_spank_-parB-mkate2* were created by amplification of *parB-mkate2* from pMK17 and *parB-gfp* from pMK11 (38, 41, 44) with primers 133 and 134. *parB-gfp* and *parB-mkate* were cloned into pMB002 using NheI and HindIII (FastDigest; Thermo Scientific), ligated with T4 ligase (Thermo Scientific), transformed into Escherichia coli DH5α, and sequenced. B. subtilis 168 *amyE*::P*_xyl_-comK* was transformed with pMB002-*parB-mkate* or pMB002-*parB-gfp*. B. subtilis 168 *amyE*::P*_xyl_-comK* P*_comg_-gfp* was created by transformation with chromosomal DNA from B. subtilis 168 P*_comG_-gfp* (21). All strains used in this study are listed in Table 7, and the primers used in this study are listed in Table 8. P_*spank*_ and P_*hyspank*_ are the promoters of plasmids pDR110 and pDR111, respectively, which were gifted to our lab by David Rudner.

**TABLE 7:** B. subtilis 168 strains used in the study

Relevant description	Genomic context	Reference
P*_xyl_-comK*	*amyE*::P_*xylR*_-P_*xylA*_*-comK*, *trpC2* Cm^r^	
P_*xyl*_-*comK*-P_*comG*_*-gfp*	*amyE*::P_*xylR*_-P_*xylA*_-*comK*-P_*comG*_-*gfp*, *trpC2* Cm^r^ Km^r^	This study
P*_xyl_-comK comFC-gfp*	*amyE*::P_*xylR*_-P_*xylA*_-*comK comFC-gfp*, *trpC2* Cm^r^ Tet^r^	This study
P_*xyl*_-*comK* P*_spank_-parB-gfp*	*amyE*::P_*xylR*_-P_*xylA*_-*comK thrC*::P_*spank*_* parB-gfp*, *trpC2* Cm^r^ Ery^r^	This study
P*_xyl_-comK parB-mkate2*	*amyE*::P_*xylR*_-P_*xylA*_-*comK thrC*::P_*spank*_* parB-mkate2*, *trpC2* Cm^r^ Ery^r^	This study
BD4477	*recA-yfp amyE*::P_*spank*_-*cfp-yjbF*, His Leu Met Cm^r ^Sp^r^	2
P*_comG_-gfp*	P_*comG*_-*gfp* Km^r^	21

**TABLE 8:** Primers used in the study

Primer	Abbreviated description^a^	Sequence
prMB013	pDG1664-ery_F	GGGAACGGTTGGAGCTAATG
prMB014	pDG1664-ery-R	TTCCGGGAACAGTGACAGAG
prMB62	U-yvyF-R	GATTTTAGAAUTGATTCTGTTTTTATGCCGATATAATC
prMB88	U-comFC-R	TTAAGCTCGAUTATGGTGTGGAAACTGGAAG
prMB89	comFC-flank-F	TGCATGCCTGUCATAGTATCCGGCACTGTTG
prMB93	tetL-R	TTCTAAAATCUTTCCTGTTATAAAAAAAGGATCAATTTTG
prMB94	P2-mcherry-F	GATCCGGATUCTGGTGGAGAAGCTGCAGCTAAAG
prMB97	P2-U-comFC-mcherry-F	ATCCGGATCUGCTTCTGATCAAGGTAAAAG
prMB100	tetL-mCherry-F	TAAGAATTCGUATGAACAGCTTATTTACATAATTCAC
prMB108	P3-gfp-dsm-R	CGAATTCTTAUTTACTTATAAAGCTCATCCATGCCGTGAGTG
133		GATCAAGCTTGAGTACTGATTAACTAATAAGGAG
134		TACTAGCTAGCGCTATCAAAAGAATCTTGC

a pDG1664, template; F, forward; R, reverse.

### Growth conditions.

The medium used was adapted from Spizizen medium (42) and was comprised of 1.8 ml distilled water, 200 µl 10× competence medium stock (0.615 M K_2_HPO_4_ ⋅ 3H_2_O, 0.385 M KH_2_PO_4_, 20% fructose [replacing glucose as the carbon source], 10 ml 300 mM Tri-Na-citrate, 1 ml 2% ferric NH_4_ citrate, 1 g casein hydrolysate [Oxoid], 2 g potassium glutamate), 10 µl 2-mg/ml tryptophan, 6.7 µl 1 M MgSO_4_. With the exception of strain BD4477 (*recA-yfp*), the strains for the nonmicrofluidics microscopy were grown in the medium containing fructose as the carbon source (2). A single colony was dropped into 2 ml of the medium, and DNA was added to 400 µl of the medium after 5 h. For the colocalization experiments using ComFC-GFP, RecA-YFP, ParB-GFP, and ParB-mKate, the total volume was scaled up to a final volume of 20 ml. For these experiments, the following conditions were used. A single colony was diluted 10^3^- to 10^5^-fold in phosphate-buffered saline (PBS) or 1× Spizizen solution to ensure that the cultures were in the exponential growth phase/early stationary phase after overnight growth, and 100 µl of this diluted single-colony solution was added to 20 ml of medium containing strain-specific antibiotics in 100-ml Erlenmeyer flasks and grown overnight at 37°C and 220 rpm. The overnight cultures were diluted to an optical density at 600 nm (OD_600_) of 0.05 in 20 ml of medium without antibiotics. The P*_xyl_-comK* strains were induced with 0.5% xylose after 4 h of growth. The P*_spank_-parB* strains were also induced with 1 mM of IPTG after 4 h of growth, and DNA was added to a concentration of 1 µg per 400 µl of the medium after 5 h. For the transformation experiment whose results are presented in Table 1, a total of 375 ng of DNA was added to 150 µl of cells. The volumes of labeled DNA in the mixture were 0.88 µl for the 100% labeled-DNA sample, 0.8 µl for 75%, 0.73 µl for 50%, 0.73 µl for 25%, and 0.57 µl for the unlabeled (0%) sample. Transformation efficiencies were calculated using the tool at https://www.sciencegateway.org/tools/transform.htm.

### PCR for labeling with fluorescein.

One microliter of 1 mM fluorescein-12-dUTP (Thermo Fisher Scientific), 2 µl dNTP mix (1 mM dATP, dCTP, and dGTP and 0.5 mM dTTP [Thermo Fisher Scientific]), 0.5 µl DreamTaq DNA polymerase (Thermo Fisher Scientific), 5 µl DreamTaq buffer, 1 µM prMB013, 1 µM prMB014, and 2 ng pDG1664 (43) in a total reaction mixture volume of 50 µl were used for fluorescein-labeling PCR. Thirty-five cycles of a standard DreamTaq PCR protocol were used. A longer extension time of 3 min was used for a 2,300-bp product. After PCR, samples were incubated for 2 h with 0.5 µl DpnI per 50-µl PCR sample (FastDigest; Thermo Fisher Scientific). PCR samples were purified using a Macherey-Nagel PCR kit. Samples were stored at −20°C. Samples were protected from light at all times. The label incorporation of fluorescein-dUTP lies between 1 and 3 pmol/µl as measured using the NanoDrop (Thermo Fisher Scientific).

### Labeling with DyLight 650/550.

The reaction conditions for DyLight labeling were the same as for fluorescein, but with 1 µl of dNTP mixture (10 mM dGTP, dCTP, and dATP and 5 mM dTTP and aminoallyl-dUTP [Thermo Fisher Scientific]). Samples were purified with a Macherey-Nagel PCR kit. The second wash step was done with 80% ethanol, and the samples were eluted with 60 µl 0.1 M NaHCO_3_, pH 9. Samples were incubated for 3 h with DyLight 650 or DyLight 550 (Thermo Fisher Scientific). Samples were purified with a Macherey-Nagel PCR kit. Labeling generally resulted in an incorporation of 1 to 2 pmol/µl as measured using the NanoDrop.

### Labeling with Alexa Fluor 5.

A BioPrime total genomic DNA-labeling module (Thermo Fisher Scientific) was used for Alexa Fluor labeling. Incorporation of Alexa Fluor 5 dNTPs was done according to the manufacturer’s protocol, except that the manufacturer’s primer solution was replaced with specific primers prMB013 and prMB014. pDG1664 was used as the template. The label incorporation lay between 5 and 9 pmol/µl as measured using the NanoDrop.

### Sample preparation.

Cultures were incubated with DNA. At the desired time point for harvesting, the samples were incubated for 10 min at 37°C with 10 U DNase I (Sigma-Aldrich) or for 40 min at 37°C with 2 mg/ml DNase I (Roche) and 1× DNase I buffer (Roche). The samples were spun down (5,000 × *g*) and washed with 1× PBS. For fixing cells (20 min at room temperature), a 2% formaldehyde solution made from paraformaldehyde dissolved in 1× PBS (pH 7.4) was used. After fixation, cells were washed with PBS and treated with DNase I as described above.

### Slide preparation.

For the time-lapse microscopy experiment, cells were immobilized using 1.5% agarose in 1× PBS, and polyacrylamide slides were made with 500 µl 40% bis-acrylamide and 1.5 ml 1× PBS or competence medium, 20 µl 10% ammonium persulfate, and 2 µl tetramethylethylenediamine (TEMED). A gene frame (Thermo Fisher Scientific or Westburg) was stuck on a glass object carrier, and the polyacrylamide was added and covered with another object carrier. The slide was left to solidify, after which the top slide was removed and the solidified gel was washed 3 times for 30 min with PBS. The gel was kept in PBS until needed and cut into smaller pieces when necessary. For the time-lapse microscopy experiment, the polyacrylamide slides were made using 1× competence medium and the slides were washed 3 times for 30 min in sterile double-distilled water and kept in 1× competence medium until needed.

### Microscopy.

Microscopy was performed on a GE Healthcare Olympus IX71 or a DeltaVision (DV) Elite microscope. For details regarding image size and exposure, see the figure legends. For the colocalization experiments, foci were considered to be colocalized when a minimum of 50% overlap of the foci occurred. Images were deconvolved with the SoftWorks imaging software. Color assignment and overlay images were created using ImageJ and saved as red/green/blue (RGB) tagged-image file format (TIFF) files. Where needed, whole images were adjusted for brightness/contrast using ImageJ.

### Microfluidics experiments.

Cultures were grown in glucose-containing competence medium as described above in “Growth conditions.” After 4 h, 0.5% xylose was added, and 1 mM IPTG for the P*_spank_-parB* constructs. After 5 h, 2 ml of the culture was spun down at maximum rpm on a standard tabletop centrifuge and the supernatant was filtered with a 30-mm, 0.45-µm Puradisc FP filter. After 5 h of growth, the culture was diluted 50× in filtered supernatant. The experiments were performed with the CellASIC ONIX microfluidic device (Merck Millipore) in a BO4A plate. The plate was primed with the medium before the start of the experiment, using the manufacturer’s specifications. The samples were loaded according to the manufacturer’s specifications. The following medium and growth conditions were used: step 1, incubation with 2.5 ng/µl DNA in 100 µl supernatant for 2 h; step 2, incubation with fresh medium containing 10 U of DNase I for 10 min; step 3, incubation with fresh medium for 1 h; and step 4, incubation for 16 h with fresh medium containing selection antibiotics. For experiments with the P_*spank*_-*parB* and P*_hyspank_-gfp* strains, 1 mM of IPTG was present in each medium for all conditions. The flow rate was 0.25 psi. Experiments were performed at 37°C. For details regarding imaging, see the figure legends. The images were deconvolved with the SoftWorks software. Analysis was done using ImageJ. For the experiment whose results are given in Table 6 and Movie S5, the determination of disappearance of foci was done by eye, and a focus was scored as disappeared when it was no longer visible for 4 frames (1 h). For the experiment whose results are given in Fig. 5, growth rate was determined by measuring cell length with the measure tool of ImageJ and calculated by linear regression, with 15 cells being followed for each sample. To determine the doubling time, 29 cells were followed, as shown in Fig. 6 and Movie S3. Movies were made with the Softworks software or with ImageJ. On average, 5 movies were analyzed for each experiment.
