# Supplementary figures and images for: Fluorescently Labeled DNA Interacts with Competence and Recombination Proteins and Is Integrated and Expressed Following Natural Transformation of Bacillus subtilis
Source: mBio. 2018 Sep 25;9(5):e01161-18. doi: 10.1128/mBio.01161-18 (PMC6156202; doi:10.1128/mBio.01161-18)

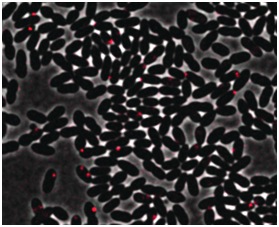

Supplement: FIG S1 [file mbo004184057sf1.tif]

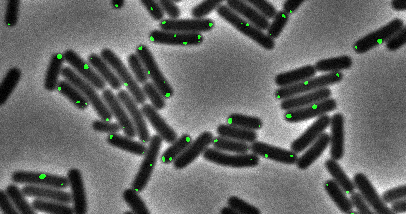

Supplement: FIG S2 [file mbo004184057sf2.tif]

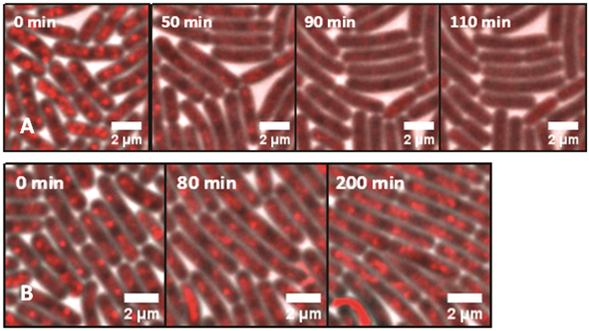

Supplement: FIG S3 [file mbo004184057sf3.tif]

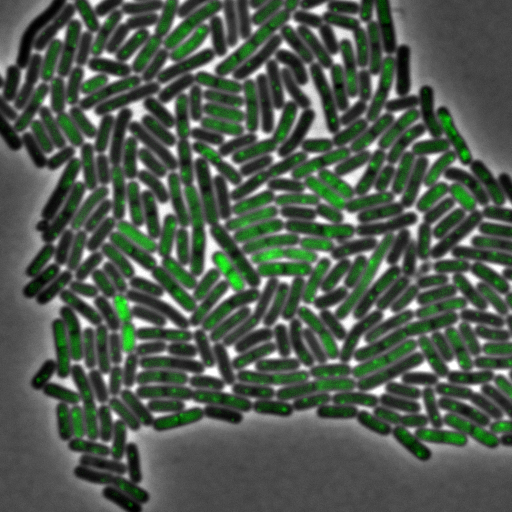

Supplement: FIG S4 [file mbo004184057sf4.tif]
