# Supplementary material for: Fluorescently Labeled DNA Interacts with Competence and Recombination Proteins and Is Integrated and Expressed Following Natural Transformation of Bacillus subtilis
Source: mBio. 2018 Sep 25;9(5):e01161-18. doi: 10.1128/mBio.01161-18 (PMC6156202; doi:10.1128/mBio.01161-18)
Supplement: TABLE S1 [file mbo004184057st1.docx]

| **DNA binding to cells during competence, tot. cells 1458** | | | |  |
| --- | --- | --- | --- | --- |
|  | **competent foci** | **competent no foci** | **non-competent foci** | **non-competent no foci** |
| **number of cells** | 262 | 304 | 42 | 850 |
| **% of cells** | 18 | 20.9 | 2.9 | 58.3 |
